# The identity of *Prunusdielsiana* (Rosaceae)

**DOI:** 10.3897/phytokeys.126.35305

**Published:** 2019-07-12

**Authors:** Baohuan Wu, Daniel Potter, Dafang Cui

**Affiliations:** 1 College of Forestry and Landscape Architecture, South China Agricultural University, Guangzhou 510642, China South China Agricultural University Guangzhou China; 2 Department of Plant Sciences, University of California, One Shields Avenue, Davis, CA 95616, USA University of California Davis United States of America

**Keywords:** *
Prunus rufoides
*, *
Prunus carcharias
*, taxonomy, typification, China

## Abstract

The valid publication date of *Prunusdielsiana* was found to be later than that of *P.rufoides*, which has been considered a synonym of *P.dielsiana*. *Prunusdielsiana* is therefore reduced to a synonym of *P.rufoides*, instead of the reverse. In addition, all previously named varieties of *Prunusdielsiana*, including var. abbreviata, var. conferta, and var. laxa, as well as *P.carcharias* are also listed as synonyms of *P.rufoides* in the present paper.

## Introduction

PrunusL.subg.Cerasus A. Gray, a group commonly known as cherries, is historically controversial in its taxonomy. As concluded by Wu et al. (2018), the taxonomy of this clade needs extensive study.

*Prunusdielsiana* C. K. Schneid. is a species widely distributed around central China and east China (Li and Bartholomew 2003). While reviewing the protologue of this species of P.subg.Cerasus, *P.dielsiana* C. K. Schneid. was found to be an invalid name. This name first appeared in Schneider’s account in 1905. Schneider (1905) proposed a description of a cherry collection, *Wilson 308*, which he determined as “*Prunusszechuanica* var.?,” indicating his uncertainty about its identification. Schneider stated that should this collection be a new species or a distinct variety of *Prunusszechuanica*, he would have proposed to name it as “*P. dielsiana*” or rather “var. *dielsiana*”. Schneider set the varietal name in bold and also indicated acceptance of the varietal status by citing only “Prunusszechuanicavar.dielsiana” in the index. Prunusszechuanicavar.dielsiana C. K. Schneid. was therefore validly published while *P.dielsiana* C. K. Schneid. was invalid.

*Prunusdielsiana* was validly published seven years later by Koehne (1912), who provided an entirely new description of the taxon. The name was ascribed to “Schneider in Fedde, Rep. Nov. Sp. I. 68 (1905)”, which should be treated as a reference to the basionym, and Koehne’s name should be considered as a new combination based on Prunusszechuanicavar.dielsiana C. K. Schneid..

Unaware of the fact mentioned above, Yü and Li (1986) published a combination, *Cerasusdielsiana* (Schneid.) Yü et Li in “*Flora Reipublicae Popularis Sinicae*”, with a direct reference to “*Prunusdielsiana* Schneider (Fedde, Rep. Nov. Sp. I. 68 1905)” rather than to the correct varietal name. Nevertheless, this reference satisfies the requirements of Art. 41.5 of International Code of Nomenclature for algae, fungi, and plants (ICN), and the errors in the basionym citation are correctable under Art. 41.6 (Turland et al. 2018). Yü et Li’s name should be recognized as a combination based on Prunusszechuanicavar.dielsiana C. K. Schneid. and should be cited as *Cerasusdielsiana* (C. K. Schneid.) Yü et Li.

Most recently, *Prunusrufoides* C. K. Schneid. was listed as a synonym of *Cerasusdielsiana* by Li and Bartholomew (2003). This is incorrect since *P.rufoides* C. K. Schneid. was validly published in 1905, while the earliest homotypic species-level synonym for *C.dielsiana* was published in 1912. Thus, Li and Bartholomew (2003) should have listed *C.dielsiana* (C. K. Schneid.) Yü et Li as a synonym of *P.rufoides* C. K. Schneid.

Three varieties have been published under *Prunusdielsiana*. Prunusdielsianavar.conferta and P.dielsianavar.laxa were described by Koehne (1912), based on specimens from western Hubei. They were thought to be different in their involucres (bracts subtending the inflorescence), with the involucres of P.dielsianavar.conferta described as erect and closed and those of P.dielsianavar.laxa described as open or sub-reflected. However, neither of these varieties was accepted as distinct in “*Flora Reipublicae Popularis Sinicae*” (Yü and Li 1986) and “*Flora of China*” (Li and Bartholomew 2003), where both were listed as synonyms of *Cerasusdielsiana*. In addition, P.dielsianavar.conferta was based on the same type as P.szechuanicavar.dielsiana, making it a later homotypic synonym that cannot be validly published under Art. 22.2 of ICN (Turland et al. 2018).

**Figure 1.**
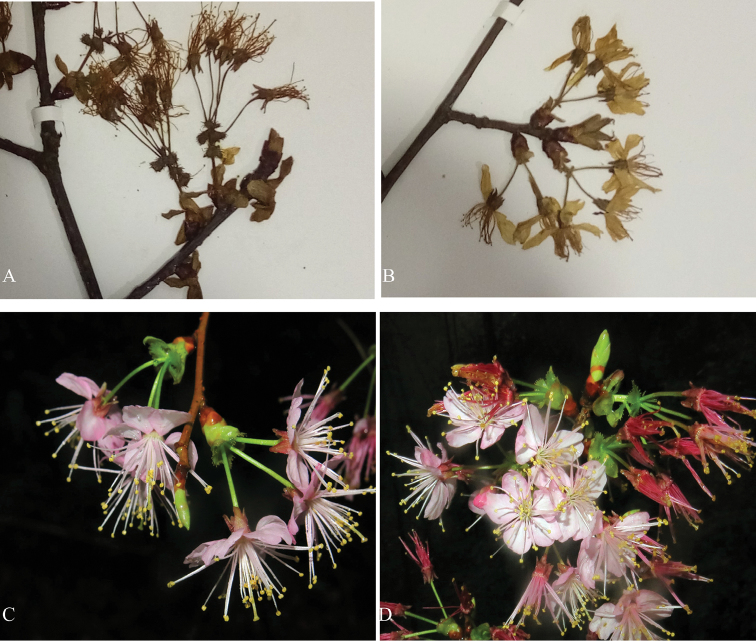
Variations of flora characters of *Prunusrufoides*. **A** (Zhou et Song 1405020, CSFI026575) **B** (Zhou et Zhou 1403107, CSFI026572) specimens collected from Hunan, Suining County, Huangsang Reserve **C, D** photos taken by Dr. H. Z. Feng, in Guangxi, Rongshui county, from the same individual.

Prunusdielsianavar.abbreviata Cardot was described by Cardot (1920) based on *Cavalerie et Fortunat 2276* collected from Guizhou (Kouy-Tcheou). He stated that this variety was similar to P.dielsianavar.conferta Koehne, but different in its narrow involucres and very short peduncles hidden in the involucres. This variety was accepted as distinct in “*Flora Reipublicae Popularis Sinicae*” (Yü and Li 1986) and “*Flora of China*” (Li and Bartholomew 2003), but we disagree with this interpretation. The distinguishing trait is rather unstable, and even in the type specimen, not all the peduncles are hidden in the involucre. As mentioned by Wang (2014), *P.dielsiana* is a highly variable species. In our field and herbarium specimen observations, leaf and floral characters, including those of the involucres and peduncles that were used to distinguish these three varieties, exhibited extensive variation (Figure 1). Therefore, we deem that none of the three previously named varieties is worthy of taxonomic recognition.

**Figure 2.**
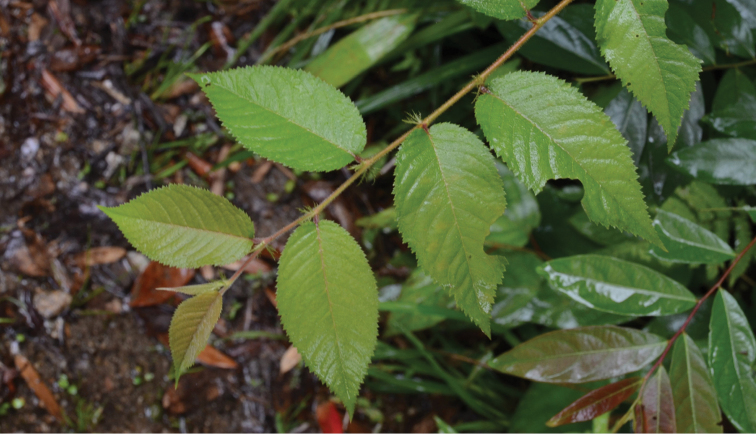
Adventitious shoot of *Prunusrufoides* C. K. Schneid. (photograph by Dr. W. Y. Zhao).

*Prunuscarcharias* was described based on a leafy branch without flowers and fruit collected from China, Chongqing, Nanchuan County (Koehne 1912), and the name is still unresolved today (Li and Bartholomew 2003). This species was considered as a member of P.sect.Microcerasus (Spach) C. K. Schneid. by Koehne (1912) because its leaf shape and serration were very similar to *P.nakaii* H. Lév. (P.japonicavar.nakaii (H. Lév.) Rehder). However, we believe that this branch must represent an adventitious shoot of *P.rufoides* (Figure 2), a phenomenon commonly observed in Nanchuan. Leaf dimorphism often occurs in P.subg.Cerasus, such that the leaves on the adventitious shoots and summer shoots may be obviously different from typical leaves of the species. Therefore it is not advisable to describe new species of this clade based solely on differences in leaf morphology, and we consider *P.carcharias* Koehne to be a synonym of *P.rufoides*.

Koehne cited three gatherings from Hubei, *Wilson 37*, *Wilson 37a* and *Wilson 68*, when describing P.dielsianavar.laxa. There are 12 sheets of these three gatherings that can be found in the Global Plant Database (JSTOR 2019), and we found that two sheets of *Wilson 37a*, both with identification slips from Koehne, were mixed species collections. One of them, HBG511107, contains a flowering branch of *P.conradinae* Koehne, while another one, US00623845, contains a leafy branch of *P.tomentosa* Thunb. Therefore, a lectotype was selected in agreement with Art. 9.3 and Art. 9.14 of ICN (Turland et al. 2018).

## Taxonomic treatment

### *Prunusrufoides* C. K. Schneid., Repert. Spec. Nov. Regni Veg. 1: 55. 1905.

Type: China, Sichuan (Szetschwan), A. Henry 5780 (E [E00011284 image!], US [US00107992 image!]).

= Prunusszechuanicavar.dielsiana C. K. Schneid., Repert. Spec. Nov. Regni Veg. 1: 68. 1905, syn. nov. ≡ *Prunusdielsiana* (C. K. Schneid.) Koehne, Pl. Wilson. (Sargent) 1(2): 243. 1912 ≡ Prunusdielsianavar.conferta Koehne, Pl. Wilson. (Sargent) 1(2): 244. 1912, nom. inval. ≡ *Cerasusdielsiana* (Koehne) Yü et Li in Fl. Reipubl. Popularis Sin. 38: 59. 1986.

Type: China, Hubei, Badong, April 1900, E. H. Wilson Veitch Exped. 308 (A [A00032048 image!], E [E00011281 image!], NY [NY00429944 image!], P [P01819046 image!]).

= *Prunuscarcharias* Koehne, Pl. Wilson. (Sargent) 1(2): 267–268. 1912, syn. nov.

Type: China, Chongqing, Nanchuan, A. von Rosthorn s. n. (holotype: B; isotype: A [A00026999 image!].

= Prunusdielsianavar.laxa Koehne, Pl. Wilson. (Sargent) 1(2): 208. 1912, syn. nov.

Type: China, Hubei, Xingshan, 1907, E. H. Wilson 68 (lectotype designated here: A [A00032051 image!]; isolectotype: A [A00032052 image!], E [E00011280 image!], HBG [HBG511108 image!], P[P01819047 image!], US [US00107951 image!]); China, Hubei, Badong, 1907, E. H. Wilson 37 (paratype: A [A00032049 image!], HBG [HBG511106 image!], US [US00623846 image!]); China, Hubei, Badong, 1907, E. H. Wilson 37a (paratype: A [A00032050 image!], HBG [HBG511107 in part, image!], US [US00623845 in part, image!]).

= Prunusdielsianavar.abbreviata Cardot, Notul. Syst. (Paris) 4(1): 29. 1920, syn. nov. ≡ Cerasusdielsianavar.abbreviata (Cardot) Yü et Li, Fl. Reipubl. Popularis Sin. 38: 59. 1986.

Type: China, Guizhou, Pingfa, 1905, Cavalerie et Fortunat, 2276 (syntype: P [P03357963 image!]); China, de la Touche 32 (syntype: E).

## Appendix 1

Additional specimens examined: **(The code of the herbaria and barcode numbers was proposed in the brackets) China**, **Chongqing**: **Beibei**, Chuanqiandui 142 (PE 00773209), Chuanqiandui 189 (PE 00773212); **Chengkou**, R. P. Farges s. n. (P 03357965, P 03357966, P 03357967, P 03357968, P 03357969, P 03357970, P 03357971, P 03357972, P 03357973, P 03357974, P 03357975, P 03357976, P 03357977, P 03357978, P 04149089, P 04167989); **Nanchuan**, Zhengyu Liu15323 (P 03358066, PE 00773218), Jihua Xiong & Zilin Zhou 90010 (PE 00773198, PE 01438524), Jihua Xiong & Zilin Zhou 90016 (IBSC 0295268, IBSC 0295269, PE 00773216, PE 01438523), Jihua Xiong & Zilin Zhou 90679 (PE 00773217), ZhongguoxibukexueYuan 2867 (PE 00773196), ZhongguoxibukexueYuan 2877 (PE 00773211). **Wushan**, T. P. Wang 10628 (PE 00773197), Guanghui Yang 57654 (PE 00773204). **Fujian**: **Ningde**, Xiangxiu Su CSH15063 (CSH CSH0120724). **Guangxi**: **Rongshui**, Yuanbaoshan-zonghe-kaochadui Y1334 (IBK IBK00226390). **Guizhou**: **Daozhen**, Anonymous 16348 (PE 00773219); **Jiangkou**, Zhisong Zhang & Chengzhong Dang 400177 (HGAS 020949, PE 01296334, PE 01296335); **Qianyang**, Xuegen Li 202823 (IBSC 0295265), Xuegen Li 202918 (IBSC 0295264), Peixiang Tan 60529 (IBSC 0295266); **Suiyang**, Kaimin Lan 90-0678 (GZAC GZAC0016518, GZAC GZAC0016519); **Yinjiang**, Mingtai An YJ-0101 (GZAC GZAC0016714). **Hubei**: Gengguo Tang 231 (IBK IBK00063118); **Lichuan**, C.T.Hwa 0331 (PE 00773201), Zhichi Ye 471 (PE 00773202). **Hunan**: **Dayong**, Hui Zhou & Dasong Zhou 16031505 (CSFI CSFI044807, CSFI CSFI044808), Hui Zhou & Dasong Zhou 16031111 (CSFI CSFI044809, CSFI CSFI044810,CSFI CSFI044811), Hui Zhou & Jinlong Luo 15032711 (CSFI CSFI045002, CSFI CSFI045004, CSFI CSFI045005); **Hongjiang**, Xuegen Li 202823 (IBSC 0295265); **longshan**, Yan Xiao & Jianjun Zhou LS-079 (CSH CSH0102519); **Liuyang**, Xu Zhang 2015033004 (CSFI CSFI044828, CSFI CSFI044830, CSFI CSFI056935); **Shaoyang**, Lindong Duan 619 (PE 01438525); **Suining**, Jianjun Zhou & Dian Zhou 1403107 (CSFI CSFI026572, CSFI CSFI026576, CSFI CSFI026577), Jianjun Zhou & Zongping Song 1404091 (CSFI CSFI026574), Jianjun Zhou & Zongping Song 1405020 (CSFI CSFI048943, CSFI CSFI026575); **Xupu**, Hengsong Liao 98 (CSFI CSFI011565, CSFI CSFI011566, CSFI CSFI011567); **Yongxing**, Jianggeng Xiao 1008 (CSFI CSFI011574); **Xinshao**, Bangyi Li 6437 (PE 01438526); **Yongshun**, HunannongxueYuan 3622 (PE 00773199). **Jiangxi**: **Fengxin**, Ceming Tan et al. 1506604 (JJF JJF00018547), Ceming Tan et al. 1506577 (JJF JJF00018548, JJF JJF00018549); **Jinggangshan**, Qiang Fan et al. JGS-1022 (SYS SYS00173104), Qiang Fan et al. JGS4004 (SYS SYS00172771), Qiang Fan et al. JGS4075 (SYS SYS00175310); **Lichuan**, Nonglinzhi 350 (JXAU JXAU0004787) Xiangxue Yang 650052 (IBSC 0295247); **Pingxiang**, Xinghua Shi 84004(JXAU JXAU0004770); **Tonggu**, Zhengming Tao 960074 (JXAU JXAU0004779); **Wuning**, Jihua Zhang TCM702 (JJF JJF00018545); **Yichun**, Wanyi Zhao et al. LXP13-10757 (SYS SYS00181906). **Sichuan**: C.T.Hwa 331 (NAS NAS00357021), C.T.Hwa 343 (NAS NAS00357022), Yang 3054 (PE 00773208); **Kangding** (Tachienlu), A. E. Pratt 807 (P 03357964); **Tianquan**, Guiling Qu 2304 (PE 00773195, PE 00773203, PE 00773207, PE 00773210); **Xuyong**, Xinfen Gao et al. HGX10091 (CDBI CDBI0226762), Xinfen Gao et al. HGX10188 (CDBI CDBI0226725). **Yunnan**: **Qiaojia**, Anonymous 19181 (PE 00773220).
